# Diagnostic and treatment factors associated with poor survival from prostate cancer are differentially distributed between regional and metropolitan Victoria, Australia

**DOI:** 10.1186/s12894-016-0172-4

**Published:** 2016-09-02

**Authors:** Rasa Ruseckaite, Fanny Sampurno, Jeremy Millar, Mark Frydenberg, Sue Evans

**Affiliations:** 1 Department of Epidemiology and Preventive Medicine, School of Public Health and Preventive Medicine, Faculty of Medicine Nursing and Health Sciences, Monash University, Melbourne, Australia; 2 Alfred Health Radiation Oncology, Melbourne, Australia; 3 Department of Surgery, Monash Medical Centre, Melbourne, Australia

**Keywords:** Prostate cancer, Regional, Clinical registry, Patterns-of-care

## Abstract

**Background:**

Men diagnosed with prostate cancer (PCa) in specific regional areas in Victoria, Australia have a poorer five-year survival rate compared to men living elsewhere in Victoria. This study aims to describe patterns-of- presentation and -care for men diagnosed with PCa in a specific regional Victorian area, and compare the outcomes with other Victorian regions.

**Methods:**

Information on consecutive men diagnosed between 2008 and 2013 was extracted from the Prostate Cancer Outcomes Registry-Victoria. Descriptive analyses summarized diagnostic and treatment patterns of the 7,204 men with PCa in the selected region (*n* = 373), metropolitan Melbourne (*n* = 2,565) and remaining areas of Victoria (*n* = 4,266) to compare risk factors, treatments and time-taken-to-treatment.

**Results:**

Men with PCa in the selected region were more likely to be diagnosed at older age (aged 68.6 *vs* 66 years in the rest of Victoria), and incidentally rather than through case-finding PSA blood tests. They were more likely to be presented with higher NCCN risk of the disease (High: 26 %, 24 % and 20.3 %; Very high/Metastasis: 11.8 %, 5.2 % and 5.7 % in the study region, metropolitan Melbourne and elsewhere in Victoria, respectively).

Men in the selected region were also more likely to have a longer time from diagnosis to treatment (on average 15–30 days longer when compared to the rest of Victoria).

**Conclusions:**

Poorer outcomes of men with PCa in this specific region might be explained by multiple factors, including clinical-, patient-, and health-system-related. This range of explanatory factors, occurring at multiple points along the pathway of diagnosis and detection, suggests that interventions to improve outcomes for PCa in regional areas such as this need to be systematic. Interventions specifically addressing any one factor in isolation are unlikely to have much effect.

## Background

Australia and New Zealand have among the highest incidence of prostate cancer (PCa) in the world with a combined age-standardised incidence rate of 104.4 cases per 100,000 [1]. Previous studies demonstrated statistically significant and increasing mortality excess for PCain regional and rural areas in Australia and New Zealand [2, 3]. Haynes et al. [4] investigated cancer survival in New Zealand and showed that survival from PCa was poor in men living distantly from primary care and cancer centres. Geographic patterns of PCa mortality and variations in access to medical care were also examined in the US by Jemal et al. and Singh et al. [5, 6]. These studies showed that men in non-metropolitan counties generally had higher death rates and incidence of late-stage disease and lower prevalence of prostate-specific antigen (PSA) screening than in metropolitan areas. Rural and urban inequalities and increased PCa mortality were explained by the variation in screening, management of the disease and access to medical services and primary care [7, 8].

There is disparity between PCa survival in regional areas compared with metropolitan areas across Australia (5-year relative survival = 87.7 % *vs* 91.4 %, *p* < 0.001) [9]. According to the Victorian Cancer Registry (VCR) data, [10] the relative survival for one regional geographic region in Victoria, defined by a Government cancer health service boundary (an Integrated Cancer Service [ICS] area) five years after the diagnosis was approximately by 7 % lower than in metropolitan Melbourne (93 %, 95 % CI 92–93 %) and amongst the lowest in regional Victoria.

Potential causes for worse outcomes in this particular ICS could include epidemiological errors (i.e. wrong classification and coding of the disease); differences in the makeup of the population (i.e. more men with PCa risk factors living in this area); differences in disease at diagnosis (i.e. men are diagnosed with more advanced disease); differences in access to treatment (i.e. less men have curative treatment); and differences in patterns of treatment (i.e. some forms of treatment not available). The aim of this study was to evaluate factors associated with poorer survival outcomes in one regional Victorian ICS and compare the outcomes with other Victorian regions.

## Methods

### Study region

The selected regional ICS (further referred to as “a study region”) covers over 18 % of Victoria’s total landmass. In 2011, the region’s estimated resident population was 270,512 and represented ~5 % of Victoria’s total population [11]. By 2026, its population is projected to increase by 21 % compared with an overall state average increase of 23 %. This region comprises of 16 health services.

### Victorian prostate cancer registry

The Victorian Prostate Cancer Registry (now termed the Prostate Cancer Outcomes Registry-Victoria, PCOR-Vic) was established in 2009 as a rapid case-ascertainment registry to monitor patterns- and quality- of-care for Victorian men diagnosed with PCa. The registry collects data on PCa cases from 38 metropolitan and regional public and private hospitals in Victoria. Based on the latest update from Victorian Cancer Registry [12], these sites account for about 70 % of incidence PCa cases in that State, and more than 10,000 men have been accrued. Registry recruitment is linked with mandatory notification of cancer status to the population-based Victorian Cancer Registry. Details of the registry, including methods for data collection, are described elsewhere [13].

### Inclusion and exclusion criteria

Men from the PCOR-Vic were included in the study if they had been diagnosed with a pathological diagnosis of PCa between August 2008 and July 2013 and were notified to the registry after the date on which the relevant hospital began contributing data. An explanatory statement, available in 12 common languages, was sent to men who were eligible to participate in the study about 9 months after they had been diagnosed. The statement invited them to participate in the PCOR-VIC and provided an opt-out option. Consent was obtained from clinicians to include all their patients into the registry. Patients were ineligible if their diagnosing or treating doctor informed the registry that they were not capable of providing consent.

### Recruitment and data collection

An explanatory statement and accompanying letter was sent to men who were eligible to participate in the study. A waiver of consent was provided to enable collection of diagnostic and treatment details on all men with PCa, who (1) died before consent could be sought; and (2) were diagnosed via a transurethral resection of the prostate (TURP) and in whom their treating clinician has requested not to contact the patient. Men were able to opt out of the registry at any time. Histopathological data were captured through hospital information systems and pathology reports. Clinical information was collected from medical records by trained data collectors. Treatments provided within 12 months of diagnosis were included in the analysis. More detailed information about the steps of data collection is provided elsewhere [13].

### Statistical analysis

The National Comprehensive Cancer Network (NCCN) risk criteria for disease progression were used to classify patients into low-, intermediate- and high-risk disease (Table 1) [14].

**Table 1:** Risk adjustment model adopted among men with PCa from clinical registries in Victoria

Variable	NCCN
Low	Clinical T1–T2a stage AND GS 2–6 AND PSA level <10 ng/mL
Intermediate	Clinical T2b–T2c stage OR GS = 7 OR PSA level 10–20 ng/mL
High	Clinical T3a stage OR GS 8–10 OR PSA level >20 ng/mL
Very high (locally advanced)	Clinical T3b–T4 Any T, N1
Metastatic	Any T, Any N, M1

NCCN, National Comprehensive Cancer Network; GS, Gleason Score; PSA, Prostate Specific Antigen

Where the clinical T category was not recorded, if the Gleason score was ≤6 and the PSA concentration was <10 ng/mL, the patient was deemed to be at low risk for disease progression. In our study for data analysis purposes PSA levels were grouped into five categories: (1) <1 ng/mL, (2) 1–4 ng/mL, (3) 4.01–10 ng/mL, (4) 10.01–20 ng/mL, and (5) >20 ng/mL. Four Gleason score categories were used in this study: (1) score <7, (2) score = 7 (3 + 4), (3) score = 7 (4 + 3), and (4) ≥8.

Treatments provided within 12 months of diagnosis were included in the analysis. The following treatment categories were used: (1) Surgery (radical prostatectomy), (2) External Beam Radiotherapy Therapy and/or high dose radiation (EBRT/EBRT + HDR), (3) EBRT + Surgery, (4) Low dose radiation (LDR), (5) HDR, (6) Brachytherapy, dose unknown (BT-Unknown), (7) Androgen Deprivation Therapy (ADT), (8) Chemotherapy, (9) Active Surveillance (AS), Watchful Waiting (WW) and (10) Others (i.e. high intensity focused ultrasound etc.). For those patients whose treatment information was not recorded, treatment type was coded as “unknown”. Active treatment was defined as any Surgery, EBRT, BT, HDR, LDR, ADT or other treatment, but excluded no active treatment option. Note that we could not reliably differentiate watchful waiting from active surveillance, and thus we have called them “no active treatment”. Where treatment information was missing (i.e. the treatment field in the registry was left blank) it was coded as ‘unknown’ rather than no active treatment. More detailed information about the treatment types is provided elsewhere [13].

Distance to primary treatment facility was calculated using the patient’s residential address postcode and the hospital location on Whereis®. The Whereis® is one of Australia’s most popular free navigation applications, which provides maps and coordinates of Australian cities, towns and travel destinations with driving directions and traveller information [15].

Descriptive statistics summarized these variables in men from the study region, metropolitan Melbourne and the rest of Victoria. Analyses of variance and *X*^*2*^ tests for independence were used to examine demographic, diagnostic and treatment characteristics and their differences. Kruskal–Wallis and Mann–Whitney *U* tests were used to compare time and distance to treatment amongst the groups. Post-hoc tests were used to evaluate pairwise differences between categories for each group of patients. Type 1 errors were controlled using the Bonferroni approach, setting the level of significance to *P* = 0.0033.

A binary logistic regression analysis was performed to assess the impact of a number of factors (both individually and together) on the likelihood of men being offered an active treatment (i.e. no AS/WW) in the selected region. In the present study, the model predicted active treatment (i.e. “active treatment” outcome was set as 1, and all other treatments as 0) from demographic characteristics and diagnostic characteristics. All individual factors and their interactions that were significantly predictive, were added to the multivariate model. Univariate analysis (step 1) for all variables and their interactions was conducted to identify significant individual predictors, which yielded six significant potential factors and interactions that might be associated with men receiving active treatment of the disease. The six category variables were then added into a multivariate model (step 2).

Stata/IC 12.1 (StataCorp, TX, USA) was used for all analyses.

## Results

A total of 10,827 men with PCa were notified to the PCOR-Vic between August 2008 and July 2013. Of these, 3,610 (33.3 %) cases were excluded from the data analysis as their diagnosis was confirmed before the commencement of the study and 13 (0.12 %) patients opted out. The records of 7,204 eligible and consented men diagnosed in three regional and one metropolitan ICS were included in the analysis. 373 (5.2 %) of these men were diagnosed in the study region, 2,565 (35.6 %) were from metropolitan Melbourne and the remaining 4,266 (59.2 %) patients were diagnosed elsewhere in Victoria.

### Demographic and diagnostic characteristics

The average and median age of PCa men in the study region was significantly higher when compared to other Victorian men (Table 2). The median (IQR) age at diagnosis of men in the study region was 68.4 (45–92.5) years compared to 66 (38–96) in Melbourne and 66 (39–95) in the rest of Victoria.

**Table 2:** Demographic and clinical characteristics of men with PCa in the study region, metropolitan Melbourne and the rest of Victoria

	Study region, *n* (%)	Metropolitan, *n* (%)	Rest of Victoria, *n* (%)	*p*
Included into the study	373 (5.2 %)	2,565 (35.6 %)	4,266 (59.2 %)	0.000
Age groups				0.000
< 55	24 (6.4 %)	293 (11.4 %)	549 (12.9 %)	
56–65	117 (31.4 %)	896 (34.9 %)	1,551 (36.4 %)	
65–75	145 (38.9 %)	909 (35.4 %)	1,632 (38.3 %)	
> 76	87 (23.3 %)	467 (18.2 %)	534 (12.5 %)	
Age (mean, SD)	68.6 (8.9)	66.7 (9.3)	65.5 (8.8)	0.000
SEIFA				0.000
Lowest 10 % (0–10 %)	70 (18.9 %)	93 (3.6 %)	217 (5.1 %)	
Lowest 11–20 %	57 (15.4 %)	68 (2.7 %)	197 (4.6 %)	
Lowest 21–30 %	9 (2.4 %)	77 (3.0 %)	232 (5.5 %)	
Lowest 31–40 %	64 (17.3 %)	127 (5.0 %)	392 (9.2 %)	
Lowest 41–50 %	113 (29.9 %)	160 (6.2 %)	293 (6.9 %)	
Highest 51–60 %	32 (8.6 %)	119 (4.6 %)	291 (6.8 %)	
Highest 61–70 %	18 (4.9 %)	208 (8.1 %)	496 (11.7 %)	
Highest 71–80 %	8 (2.2 %)	325 (12.7 %)	546 (12.8 %)	
Highest 81–90 %	1 (0.3 %)	747 (29.0 %)	907 (21.2 %)	
Highest 10 % (91–100 %)	1 (0.3 %)	641 (25.0 %)	695 (16.2 %)	
Type of hospital				0.000
Private	47 (12.6 %)	1,390 (54.2 %)	2,822 (66.2 %)	
Public	326 (87.4 %)	1,175 (45.8 %)	1,444 (33.8 %)	
Method of diagnosis				0.000
TRUS	285 (76.4 %)	2,081 (81.1 %)	3,919 (91.9 %)	
TURP	77 (20.7 %)	416 (16.2 %)	291 (6.8 %)	
Other	11 (2.9 %)	68 (2.7 %)	56 (1.3 %)	
PSA (ng/mL)				0.000
< 4	45 (12.1 %)	427 (16.6 %)	688 (16.1 %)	
4.01–10	176 (47.2 %)	1,337 (52.1 %)	2,408 (56.4 %)	
10.01–20	74 (19.8 %)	395 (15.4 %)	679 (15.9 %)	
> 20.01	72 (19.3 %)	301 (11.7 %)	426 (10.0 %)	
Unknown	6 (1.6 %)	105 (4.1 %)	65 (1.5 %)	
Gleason score				0.000
< 7	155 (41.6 %)	851 (33.2 %)	1,641 (38.5 %)	
7 (3 + 4)	69 (18.6 %)	736 (28.7 %)	1,306 (30.6 %)	
7 (4 + 3)	41 (10.9 %)	394 (15.4 %)	535 (12.6 %)	
≥ 8	103 (27.6 %)	577 (22.5 %)	782 (18.3 %)	
Unknown	5 (1.3 %)	7 (0.3 %)	2 (0.0 %)	
NCCN risk group				0.000
Low	73 (19.6 %)	478 (18.6 %)	911 (21.4 %)	
Intermediate	113 (30.3 %)	1,064 (41.5 %)	1,887 (44.2 %)	
High	97 (26.0 %)	616 (24.0 %)	867 (20.3 %)	
Very high/Metastatic	44 (11.8 %)	133 (5.2 %)	245 (5.7 %)	
Unknown	46 (12.3 %)	274 (10.7 %)	356 (8.3 %)	

Men diagnosed in the region had lower rates of socio-economic advantage using Socio-Economic Advantage and Disadvantage (SEIFA) categories [16], with more than half the men (54 %) in the study region from the no more than the 4^th^ decile of SEIFA scores whereas, for example, in the men from metropolitan regions more than half (54 %) were in the *highest* two deciles, with fully 25 % in the highest decile of SEIFA scores. The SEIFA score is a composite of a number of average measures within an area including equivalent household income, occupancy type, level of educational attainment, level of employment/unemployment, occupational skill level, crowding, car ownership, marital status, housing and income support.

In addition the vast majority (87.4 %) of men in the study region was treated in public hospitals, which was different from metropolitan Melbourne and the rest of Victoria; men in the study region were more than four times less likely to be treated in a private hospital than men in metropolitan Victoria or other regional areas in Victoria.

The vast majority of men in the study region were diagnosed via TRUS; although a significantly higher percentage of new diagnoses was detected via TURP when compared to metropolitan Melbourne or other Victorian regions (20.7 %, 16.2 % and 6.8 % respectively).

When compared to the other regions, the study region also presented with a higher proportion of men with elevated levels of PSA (>20 ng/mL) at diagnosis (19.3 %, 11.7 % and 10 % respectively). Men in the study region were also more likely to be diagnosed with a higher Gleason score (8–10) than those diagnosed elsewhere in Victoria (27.6 %, 22.5 % and 18.3 % respectively) and presented with higher NCCN risk of the disease (High: 26 %, 24 % and 20.3 %; Very high/Metastasis: 11.8 %, 5.2 % and 5.7 % in the study region, metropolitan Melbourne and elsewhere in Victoria, respectively).

### Treatment characteristics

Treatment modalities, time to the initial treatment and travel distance are summarized in the Table 3. A significantly lower proportion of men with a low risk disease (19.2 %) in the study region were treated with a radical prostatectomy when compared to metropolitan Melbourne (29.7 %) and the rest of Victoria (43.9 %). Men in this area had to travel a longer distance (median [IQR] of 31.6 [18.9-69.3] km) to their health services provider, compared to those treated in metropolitan Melbourne (11.8 [6.2-25.9] km), or the rest of Victoria (21.6 [9.1-55.5] km).

**Table 3:** Treatment modalities, stratified by NCCN risk group, of men with PCa in the study region, metropolitan Melbourne and the rest of Victoria

NNCCN Risk group	Low*			Intermediate*			High*			Very high/Metastatic*		
	Study region, *n* (%)	Metropolitan, *n* (%)	Rest of Victoria, *n* (%)	Study region, *n* (%)	Metropolitan, *n* (%)	Rest of Victoria, *n* (%)	Study region, *n* (%)	Metropolitan, *n* (%)	Rest of Victoria, *n* (%)	Study region, *n* (%)	Metropolitan, *n* (%)	Rest of Victoria, *n* (%)
No treatment/AS/WW	46 (63.0 %)	244 (51.0 %)	337 (37.0 %)	36 (31.9 %)	161 (15.1 %)	201 (10.7 %)	8 (8.2 %)	45 (7.3 %)	44 (5.1 %)	1 (2.3 %)	4 (3.0 %)	3 (1.2 %)
Surgery (Prostatectomy)	14 (19.2 %)	142 (29.7 %)	400 (43.9 %)	31 (27.4 %)	543 (51.0 %)	1,104 (58.5 %)	13 (13.4 %)	168 (27.3 %)	313 (36.1 %)	2 (4.5 %)	9 (6.8 %)	21 (8.6 %)
EBRT/EBRT + HDR	5 (6.8 %)	33 (6.9 %)	56 (6.1 %)	23 (10.4 %)	161 (15.1 %)	345 (18.3 %)	35 (36.1 %)	233 (37.8 %)	311 (35.9 %)	11 (25.0 %)	60 (45.1 %)	109 (44.5 %)
EBRT + Surgery	1 (1.4 %)	5 (1.0 %)	26 (2.9 %)	3 (2.7 %)	78 (7.3 %)	108 (5.7 %)	5 (5.2 %)	60 (9.7 %)	96 (11.1 %)	0 (0.0 %)	6 (4.5 %)	9 (3.7 %)
LDR	5 (6.8 %)	22 (4.6 %)	69 (7.6 %)	7 (6.2 %)	53 (5.0 %)	68 (3.6 %)	0 (0.0 %)	0 (0.0 %)	1 (0.1 %)	1 (2.3 %)	0 (0.0 %)	0 (0.0 %)
HDR	0 (0.0 %)	0 (0.0 %)	1 (0.1 %)	1 (0.9 %)	6 (0.6 %)	3 (0.2 %)	0 (0.0 %)	1 (0.2 %)	1 (0.1 %)	1 (2.3 %)	0 (0.0 %)	1 (0.4 %)
BT-Unknown	1 (1.4 %)	8 (1.7 %)	5 (0.5 %)	0 (0.0 %)	8 (0.8 %)	6 (0.3 %)	0 (0.0 %)	1 (0.2 %)	2 (0.2 %)	0 (0.0 %)	0 (0.0 %)	0 (0.0 %)
ADT	0 (0.0 %)	3 (0.6 %)	2 (0.2 %)	9 (8.0 %)	13 (1.2 %)	23 (1.2 %)	34 (35.1 %)	92 (14.9 %)	79 (9.1 %)	23 (52.3 %)	41 (30.8 %)	84 (34.3 %)
Chemotherapy	0 (0.0 %)	0 (0.0 %)	0 (0.0 %)	0 (0.0 %)	0 (0.0 %)	0 (0.0 %)	1 (1.0 %)	1 (0.2 %)	1 (0.1 %)	4 (9.1 %)	5 (3.8 %)	13 (5.3 %)
Other	0 (0.0 %)	5 (1.0 %)	4 (0.4 %)	0 (0.0 %)	6 (0.6 %)	2 (0.1 %)	0 (0.0 %)	0 (0.0 %)	1 (0.1 %)	0 (0.0 %)	1 (0.8 %)	0 (0.0 %)
Not known	1 (1.4 %)	16 (3.3 %)	11 (1.2 %)	3 (2.7 %)	35 (3.3 %)	27 (1.4 %)	1 (1.0 %)	15 (2.4 %)	18 (2.1 %)	1 (2.3 %)	7 (5.3 %)	5 (2.0 %)
Total	73 (100 %)	478 (100 %)	911 (100 %)	113 (100 %)	1,064 (100 %)	1,887 (100 %)	97 (100 %)	616 (100 %)	867 (100 %)	44 (100 %)	133 (100 %)	245 (100 %)
Median (IQR) days to initial treatment	N/A	N/A	N/A	101 (56–191)	71.5 (44–119)	61 (39–103)	57 (34–90)	49.5 (31–84.25)	43 (27–65)	27 (6–67.5)	27 (9–58.5)	19.5 (7–50)
Median (IQR) km to treating hospital	31.6 (18.9-69.3)	11.8 (6.2-25.9)	21.6 (9.1-55.5)	56.4 (23.1-114.9)	13.1 (7.2-27.4)	20.7 (10–51.4)	42.3 (19–82.7)	11.7 (6.2-25.7)	20.8 (9.8-61.3)	39.4 (19.2-80.7)	11.4 (5.5-24.7)	25.6 (11.2-71.6)

*p < 0.05

By comparing men in the same “risk group” across regions, we can to some extent adjust for difference in management patterns overall that might be influenced by the more advanced higher grade disease seen in the region, by comparing more “like with like”. In the risk group in which attempts at curative treatment are most clearly indicated, the intermediate risk group, men had different management patterns. There were a significantly lower proportion of men in the study region - nearly half as frequent - who undertook a prostatectomy [(27.4 *vs* 51.0 % *vs* 58.5 %). EBRT treatment as also at least a third less frequent (10.4 % *vs* 15.1 % *vs* 18.3 %) when compared to metropolitan Melbourne or the rest of Victoria, even within this identical intermediate risk group. As a consequence, there was also a significantly higher proportion of men (more than twice as likely) did not receive active treatment in this particular region (31.9 % *vs* 15.1 % *vs* 10.7 %). For these men, the median [IQR] amount of time between diagnosis and treatment in the study region was notably longer: 101 (56–191) *vs* 71.5 (44–119) *vs* 61 (39–103) days in metropolitan Melbourne and the rest of Victoria respectively. Men with intermediate risk PCa in the study region also needed to travel more than four times longer distances to their treating institution (56.4 [23.1-114.9] km) than those in metropolitan Victoria.

In a high-risk cancer group, 13.4 % of men undertook a prostatectomy in the study region, significantly less (less than half as common) than in metropolitan Melbourne (27.3 %) or in the rest of Victoria (36.1 %). There men were more than twice as likely to be treated with androgen deprivation (ADT), 35.1 %, compared to 14.9 % in metropolitan Melbourne and 9.1 % in the rest of Victoria. A median [IQR] amount of time between diagnosis and treatment was 57 [34–90] days, significantly longer than in metropolitan Melbourne (49.5 [31–84.25] days) or in the rest of Victoria (43 [27–65] days). Men in the selected ICS travelled a median [IQR] of 42.3 [19–82.7] km, almost four-time further than those treated in metropolitan Melbourne (11.7 [6.2-25.7] km), or twice as far as in the rest of Victoria (20.8 [9.8-61.3] km).

In a very high/metastatic risk group patterns-of- care were similar as in other groups, with more patients in the study region (52.3 %) being treated with ADT when compared to 30.8 % in metropolitan Melbourne, or 34.3 % in the rest of Victoria. The median [IQR] amount of time to the first treatment in the study region was 27 [6–67.5] days, 27 [9–58.5] in metropolitan Melbourne and 19.5 [7–50] elsewhere. Men needed to travel the median [IQR] distance of 39.4 [19.2-80.8] km to their treatment institution, longer than in metropolitan Melbourne (11.4 [5.5-24.7] km) or in the rest of Victoria (25.6 [11.2-71.6] km).

### Regression analysis of factors determining treatment type

Table 4 summarizes the contributions of factors in the univariate and multivariate model to men receiving active treatment.

**Table 4:** Significant factors associated with the likelihood of active treatment in men with PCa (NS – Not Significant)

	Univariate model		Multivariate model	
Factors	Odds Ratio	CI at 95 %	Odds Ratio	CI at 95 %
Region				
Selected Region (Ref)	1		1	
Metropolitan	1.53	1.21-1.94	1.89	1.35-2.64
Rest of Victoria	2.21	1.74-2.78	2.59	1.86-3.61
Age				
< 55 (Ref)	1		1	
56-65	0.76	0.61-0.94	0.62	0.47-0.80
65-75	0.75	0.61-0.93	0.41	0.31-0.53
> 76	0.41	0.33-0.52	0.13	0.09-0.17
Type of hospital				
Private (Ref)	1		1	
Public	1.52	1.34-1.72	1.44	1.22-1.69
Method of diagnosis				
TRUS (Ref)	1		1	
TURP	0.17	0.15-0.20	0.22	0.17-0.28
Other	1.44	0.85-2.44	0.79	0.35-1.76
NCCN Risk				
Low (Ref)	1		1	
Intermediate	5.03	4.34-5.84	6.12	5.16-7.25
High	11.48	9.12-14.45	27.69	20.81-36.87
V.high/Metastatic	38.85	19.15-78.82	138.39	65.32-293.22
Region × NCCN Risk				
Selected Region × Low (Ref)	1		NS	NS
Metropolitan × Intermediate	3.61	2.97-4.38	NS	NS
Metropolitan × High	8.16	5.94-11.23	NS	NS
Metropolitan × V.high/Metastatic	20.75	7.64-56.42	NS	NS
Rest of Victoria × Intermediate	5.39	4.53-6.43	NS	NS
Rest of Victoria × High	12.04	8.75-16.55	NS	NS
Rest of Victoria × V.high/Metastatic	51.92	16.56-162.79	NS	NS

A full multivariate model containing all six category variables was statistically significant, *χ*^2^(17, *N* = 6,528) =1491,869 p < 0.05) indicating ability to distinguish between men with PCa who had active treatment (*N* = 5,751) *vs* those with no active treatment. The model explained between 21 % (Cox and Snell R Square) and 34 % (Nagelkerke R Square) of the variance in treatment type.

When compared to the selected region, men diagnosed in metropolitan areas had nearly twice the odds of receiving active treatment (OR = 1.89, 95 % CI, 1.35-2.64), and those in the rest of Victoria were even at higher odds, OR = 2.59, 95 % CI, 1.86-3.61). Men older than 75 years of age were nearly 90 % less likely to receive active treatment, compared to younger men of 55 years or less, OR = 0.13, (95 % CI, 0.09-0.17). Men with PCa were also more likely to receive an active treatment in public hospitals, OR = 1.44, (95 % CI, 1.22-1.69).

Those men whose diagnosis was detected via TURP were less likely to receive an active treatment, OR = 0.22, (95 % CI, 0.17-0.28) than men diagnosed via TRUS. When combined with other factors, higher NCCN Risk levels were also associated with an increased likelihood of men receiving an active treatment. For example, men with v.high/metastatic disease had extremely high odds of receiving active treatment than men diagnosed with low risk disease, OR = 138.39, (95 % CI, 65.32-293.22).

Univariate model also revealed, that compared to men in the selected region diagnosed with low risk disease, those in the other areas, and at higher risk of the disease were more likely to receive an active treatment.

## Discussion

Higher death rates from prostate (and other) cancers occur in patients from non-metropolitan regions. The population-based PCOR-Vic has enabled more precise identification of the underlying factors in one selected ICS in regional Victoria. We found that men in the study region were startlingly lower in measures of socio-economic advantage. They were older. A higher percentage of newly PCa cases in the study region (almost a fifth) were detected during TURP, a procedure performed to alleviate symptoms of urinary obstruction and not done primarily usually because PCa. Although TURP is generally not used to detect PCa anymore, as it makes a poor tool for early cancer detection [17], it used to be a common mean of detection of PCa before PSA screening was introduced.

We also found that men in the study region presented with a significantly higher level of PSA levels and Gleason score at diagnoses when compared to other Victorian regions, which supports previous research findings that men in rural and regional areas are more likely to have elevated PSA and Gleason score [18–21]. Overall, men in the study region presented for treatment at a more advanced disease stage when compared to the rest of Victoria, similarly to the findings reported by Baade and Coory, [2, 3] showing that men living in regional areas tend to be diagnosed at advanced stages of the disease. A possible reason for higher rates of a more advanced disease in the study region could possibly be due to issues of high PSA testing rates in metropolitan areas [2, 18].

Men in the study region, in the low and intermediate disease groups, were significantly less likely to undergo active treatment. There is emerging evidence that many men with early stage disease are appropriately managed with AS rather than having active invasive treatment [22, 23]. Yet, since the rates of no treatment are higher than the rates of no treatment in metropolitan Victoria, particularly in the intermediate risk group (where AS would not usually be recommended), it may be that higher rates of non-treatment represent a significant proportion of men diagnosed in the region are not having treatment as would be appropriate, rather than being placed on systematic program of active follow-up and surveillance as would be done if they were on AS. Variability in treatment patterns in different regions could also potentially reflect the managing clinicians’ treatment preferences, perhaps different in the region than other parts of Victoria, since this factor is known to be a strong determinant of the treatment received [24, 25].

There was a notable time delay between diagnosis and treatment in men with intermediate/very high risk disease in the study region when compared to the rest of Victoria. While reasons for such delay are unknown - the findings may reflect a preference by their treating doctor to manage the disease conservatively or that men themselves are more reluctant to take up the option for active treatment than in other areas of Victoria - delays of more than 30–60 days can adversely affect pathological outcomes in men in these risk groups treated with radical prostatectomy [26].

Men in the study region also travelled much longer distances for their treatment, which could have influenced their choice of treatment and determined their health outcomes. Previous studies showed that prognosis and outcomes from PCa are known to be worse for men living in rural areas and needing to travel longer distances to their treatment providers [4, 27]. Longer time and distance required to travel could act as an economic and financial barrier in men’s treatment choice, which may potentially explain why, compared with metropolitan regions, there are more men in the study region with high risk disease but who did not receive active treatment. The disadvantaged socio-economic category for many men in the region could be imagined to worsen barriers caused by distance.

As suggested by Ng et al. [28], the reasons for such discrepancies as we noted in treatment and outcomes between metropolitan and regional areas could possibly be explained by clinical-, patient-, and health-system-related factors. Potential patient-related barriers to PCa care could include not consulting a general practitioner (GP) in a timely manner [29], a lack of desire for treatment due to a ‘I will be OK’ attitude, or financial/time barriers if the care is only available outside the patient’s regional area, thus requiring considerable time and travel expenses [15]. Urban–rural disparities in PCa incidence and mortality may arise from differences in demographic and socioeconomic characteristics of these two groups that may influence access to and utilization of diagnostic and treatment services [30].

It is not surprising that men in regional areas in Australia face distinct health issues because of their location, work, and lifestyle. There is evidence that these men are more likely to experience chronic health conditions and have more risk factors for serious disease than their metropolitan counterparts. For example, epidemiological studies of Begg and Beard have demonstrated that rural dwellers are more likely than metropolitan dwellers to report daily smoking and risky drinking behavior, [31, 32] are less likely to possess an adequate level of health literacy and have higher mortality rates from injury, cardiovascular disease and diabetes [33].

The other potential reason could be a limitation on GPs referral choice, and issues concerning the quality of care due to potential for impediments to professional development of regional doctors and specialists [28]. It may be that specialists, diagnostic equipment and other medical technology, and operating rooms are much more restricted in the regional areas. Internationally, patients with cancer are increasingly being managed by multidisciplinary teams, allowing the use of varying professional skills with numerous benefits for the patient [34] and this approach is promoted by the Victorian Department of Health and Human Services. A lack of uro-oncology multidisciplinary meetings in the selected study region could also be associated with poorer outcomes in men with PCa [16].

The Australian and Victoria Governments as well as community groups have been determined to allocate resources to reduce inequity in health outcome [35]. This highlights the importance of our work. Our study has demonstrated that there are interlinked chain of factors that are associated with the poorer outcomes: the men are older, they are startlingly more likely to be in disadvantaged socio-economic groups, the live much further away from specialist health care providers, they are much more likely to be diagnosed “by accident”, as it were, and then they wait much longer for treatment. They have more advanced and more aggressive disease when they do get diagnosed; but even adjusting for this, they are much more likely not to receive any attempt at curative treatment, and much more likely to be treated in a public hospital. The health services in the region have fewer services available for the care of men with PCa. Hitherto, work has established the disparities in outcome in regional areas, but the causes were mostly supposition in the absence of data. Registries provide the data on the factors associated with these outcomes.

The major strength of this study is the use of clinical registry data, containing a detailed diagnosis and treatment information of the patients with PCa. The PCOR-Vic enables rapid and reliable ascertainment of patterns of care and quality of life data of men diagnosed with PCa and reports back to treating clinicians in regional and metropolitan Victoria [13]. However, some limitations to this study also need to be noted. Firstly, there were a relatively small number of PCa men in the study region. Another important limitation is that we did not seek to identify reasons why men received no treatment within 12 months of diagnosis in the study region. It may be that they were receiving AS, had decided not to pursue active curative treatment, were awaiting therapy on the basis of their PSA level taken at 12 months, or were inadequately managed. Finally, we were unable to investigate other factors, such as obesity, smoking habits or alcohol consumption in the study region as the registry does not collect such information.

This study has identified a number of key areas of difference in the study region. Identifying these differences allows logical plans for where the effort needs to be made to enable improvement, and where more information is needed. The findings of this study have been shared with health service providers and policy-makers in the study region. They have an on-going strategy to address many of the key points identified, to better provide health care services for men diagnosed with PCa in the region. A project is underway to better understand factors associated with why men are presenting with more advanced PCa disease than in other areas of Victoria. This multifaceted project will involve patients living in the region and their GPs. It is important that we understand reasons for this so that interventions can be targeted to areas of greatest need.

## Conclusions

The PCOR-Vic has enabled for the first time clarity around the factors associated with PCa in an Australian regional location, documenting many structure, process or outcome measurements where the location performs worse than other metropolitan or regional areas in Victoria. It suggests plans to improve outcomes need to be made on a systematic basis. Registries such as the PCOR-Vic provide vital on-going insight on how resources are best allocated to improve the lot of men diagnosed with PCa.

## Abbreviations

ADT: Androgen deprivation therapy
AS: Active surveillance
BT: Brachytherapy
DHHS: Department of health and human services
EBRT: External beam radiation therapy
GP: General practitioner
HDR: High-dose rate brachytherapy
ICS: Integrated cancer service
IQR: Interquartile range
LDR: Low-dose rate brachytherapy
NCCN: National comprehensive cancer network
OR: Odds ratio
PCa: Prostate cancer
PCOR-Vic: Prostate cancer outcomes registry – Victoria
PSA: Prostate-specific antigen
SEIFA: Socio-economic index of advantage and disadvantage
TRUS: Transrectal ultrasonography of the prostate
TURP: Transurethral resection of the prostate
WW: Watchful waiting
